# More than just talk: the framing of transactional sex and its implications for vulnerability to HIV in Lesotho, Madagascar and South Africa

**DOI:** 10.1186/1744-8603-7-34

**Published:** 2011-09-30

**Authors:** Kirsten Stoebenau, Stephanie A Nixon, Clara Rubincam, Samantha Willan, Yanga ZN Zembe, Tumelo Tsikoane, Pius T Tanga, Haruna M Bello, Carlos F Caceres, Loraine Townsend, Paul G Rakotoarison, Violette Razafintsalama

**Affiliations:** 1 International Center for Research on Women, Washington, D.C. (20036), USA; 2 Department of Physical Therapy, University of Toronto, Toronto (M5G 1V7), Canada; 3 Department of Social Policy, London School of Economics and Political Science, London (WC2A 2AE) UK; 4 Health Economics and HIV Research Division (HEARD), University of KwaZulu-Natal, Durban (X54001) South Africa; 5 Health Systems Research Unit, Medical Research Council, Cape Town (7505) South Africa; 6 Department of Development Studies, National University of Lesotho, Maseru, (P.O. Roma 180) Lesotho; 7 Department of Social Work/Social Development, University of Fort Hare, Alice (X1314) South Africa; 8 Centre for Rural Development, Walter Sisulu University, Mthatha, (5100) South Africa; 9 Heredia Instituto de Estudios en Salud, Sexualidad y Desarrollo Humano, Universidad Peruana Cayetano, Lima (18) Peru; 10 Department of Sociology and Anthropology, University of Antananarivo, Antananarivo (101), Madagascar; 11 Malagasy Socio-Consulting and Communication, Antananarivo (101) Madagascar

**Keywords:** transactional sex, discourse, HIV vulnerability, HIV risk, South Africa, Madagascar, Lesotho, globalization, qualitative research

## Abstract

**Background:**

'Transactional sex' was regarded by the mid-1990s as an important determinant of HIV transmission, particularly in sub-Saharan Africa. Little attention has been paid to what the terms used to denote transactional sex suggest about how it is understood. This study provides a nuanced set of descriptions of the meaning of transactional sex in three settings. Furthermore, we discuss how discourses around transactional sex suggest linkages to processes of globalization and hold implications for vulnerability to HIV.

**Methods:**

The analysis in this article is based on three case studies conducted as part of a multi-country research project that investigated linkages between economic globalization and HIV. In this analysis, we contextualize and contrast the 'talk' about transactional sex through the following research methods in three study sites: descriptions revealed through semi-structured interviews with garment workers in Lesotho; focus groups with young women and men in Antananarivo, Madagascar; and focus groups and in-depth interviews with young women and men in Mbekweni, South Africa.

**Results:**

Participants' talk about transactional sex reveals two themes: (1) 'The politics of differentiation' reflects how participants used language to demarcate identities, and distance themselves from contextually-based marginalized identities; and (2) 'Gender, agency and power' describes how participants frame gendered-power within the context of transactional sex practices, and reflects on the limitations to women's power as sexual agents in these exchanges. Talk about transactional sex in our study settings supports the assertion that emerging transactional sexual practices are linked with processes of globalization tied to consumerism.

**Conclusions:**

By focusing on 'talk' about transactional sex, we locate definitions of transactional sex, and how terms used to describe transactional sex are morally framed for people within their local context. We take advantage of an opportunity to comparatively explore such talk across three different study sites, and contribute to a better understanding of both emerging sexual practices and their implications for HIV vulnerability. Our work underlines that transactional sex needs to be reflected as it is perceived: something very different from, but of at least equal concern to, formal sex work in the efforts to curb HIV transmission.

## Introduction

For public health researchers, 'transactional sex' or sex for gift exchange that is understood outside of formal prostitution or sex work by its participants, was regarded by the mid-1990s as an important determinant of HIV transmission, particularly in sub-Saharan Africa ([1-3]). However, there remain a number of questions as to the meaning of and motivation for transactional sex. Answering such questions may play an important role in improving HIV prevention efforts in sub-Saharan Africa. In this paper, we contribute to better understanding the meaning of transactional sex by focusing on everyday 'talk' about transactional sex for both transactional sex participants and observers in three countries- Lesotho, Madagascar and South Africa. By contrasting what the terms used to denote transactional sex suggest about its role in various local contexts, we illustrate how examining local constructions of transactional sex reveals implications for sexual health risk. In choosing to focus on the everyday talk around transactional sex, this study builds on similar work that has examined how casual conversation depicts social phenomena [4-6].

## Background

### Discourses of transactional sex in sub-Saharan Africa

'Transactional sex,' as a concept, became relevant to HIV prevention efforts as repeated studies demonstrated HIV prevalence rates were higher among commercial sex workers than others. Concern then developed around more informal forms of sex for material exchange alongside the related issue of 'multiple concurrent partnerships' ([2,7,3]). By transactional sex, we are referring to the exchange of gifts (material, monetary) for sex framed outside of prostitution or sex work by those who participate in the exchange. As Mark Hunter explained in an article entitled "The materiality of everyday sex"[8]:

Transactional sex has a number of similarities to prostitution. ...[but] Transactional sex differs in important ways: participants are constructed as "girlfriends" and "boyfriends" ... and the exchange of gifts for sex is part of a broader set of obligations that might not involve a predetermined payment. (page 100-101, 2002).

Transactional sex relationships have been shaped by a number of economic, social and political conditions over time that have become, more often than not, dichotomized in the literature as motivated by either 'survival' or 'consumption.' Most conventionally within the context of highly unequal gendered power and position, very poor women portrayed as victims resort to 'survival sex' to acquire basic needs ([9-12]). More recently, a counter discourse has emerged that describes relatively less poor young women, within the same gendered constraints, as active agents seeking men's company in order to access consumer items, and a 'modern' lifestyle. The latter discourse is often rooted within cultural and economic processes of globalization. In this conceptualization, consumption becomes a means by which to access 'social power' ([13-17,8]). Most recently, researchers have moved away from the dichotomy to emphasize the nuance of such exchanges, the limits of women's agency, and the importance of contextualizing both notions of survival and consumption [18-20,15].

It is also important to acknowledge that despite the rather recent emergence of the term 'transactional sex,' the notion of material gift exchange for sex had already been in the anthropological record for decades. Anthropologists have observed that dynamic and diverse informal sexual exchange relationships exist which are not captured by Western notions of prostitution ([21-25]) (see [26] for a comprehensive review). Furthermore, these relationships are rooted in historical, socio-cultural and gendered practices, subject to reproduction and transformation. In the context of sub-Saharan Africa, for example, it has been recently argued that transactional sex may now serve to either redistribute wealth as bridewealth did in the past, or to display wealth as polygyny did [19]. Transactional sex provides the modern equivalent to these more traditional practices, rather than display wealth through the number of wives one can support, or the price of the bridewealth that can be paid, now a man can demonstrate his wealth through the number of girlfriends he can support. It has been further argued that in the rural Malawian context, wealthy men are almost socially obligated to engage in multiple, transactional sex relationships in order to share their wealth as an extension of patron-client notions of citizenship [27]. Reflecting back on the link to HIV, transactional sex is often implicit in descriptions of 'multiple concurrent partnerships.' When describing their motivations for such relationships, (especially women) respondents often point to material and/or monetary incentives for engaging with multiple sexual partners. Women reflect most on the financial benefits to having multiple boyfriends. (e.g. [28,11,29]). As these practices are shaped and transformed, so too is the language with which to identify and characterise them.

#### The Value of Studying Transactional Sex 'Talk'

Individuals who engage in transactional sex have evolved novel ways of referring to these practices. These phrases and expressions frame both the positive and negative aspects of the practice. As Leap and Boellstorff observed: "If there are sexual cultures then there must be sexual languages, that is, modes of describing, expressing, and interrogating the ideologies and practices relevant to the sexual culture(s)" ([30]: 12).

These 'sexual languages' serve a variety of productive purposes for observers of and participants in transactional sex relationships. Klesse (2006) refers to "the politics of differentiation," that individuals participating in, and observing certain kinds of sexual partnerships use to draw distinctions between *acceptable *or *desirable *behaviours and their alternatives. For Klesse's study of non-monogamy in the United Kingdom, individuals employed these tactics to distinguish 'polyamory' - multiple sexual partnerships where love is of central importance - from 'other' forms of non-monogamy, namely promiscuity, casual sex, and 'swinging' [31].

Research has begun to explore the linkages between globalization, transactional sex and vulnerability to HIV. However, there has yet to be an inquiry into this nexus that locates discourses around transactional sex as the object of study. Focusing on talk offers the opportunity to reveal nuances and complexities about social phenomena that may otherwise be eclipsed. In reviewing the literature that describes different terms denoting transactional sex, we identified three main productive purposes of everyday talk about transactional sex.

First, we observe that 'talk' has been used *to differentiate between informal transactional sex and commercial sex work*. Embedded in this distinction are moral judgements about appropriate versus inappropriate sexual behaviour for different groups. Most commonly, words and phrases are deliberately used to differentiate women who engage in professional commercial sex work from those who engage in other forms of reciprocal exchanges involving sex. Women in Wojcicki's (2002) study in Soweto and Hammanskraal, South Africa, use the term *ukuphanda*, a Zulu verb that loosely means "to try to get money," to distinguish between informal transactional sex and more formalized sex work (*marhosha *or *matekatse*) ([32]: 340). Stoebenau found that women who practiced informal sexual exchange in nightclubs in Antananarivo, Madagascar self-identified as going out to '*mitady vady vazaha*' (look for foreign husbands), in a deliberate effort to distance themselves from those women they identified as *mpivaro-tena *(prostitutes) [33]. In Lesotho, Anthropologists have documented a distinction between two sexual exchange relationship categories: *bonyatsi *and *botekase *(beginning in the 1970's). The former denotes relatively socially acceptable, long-term, extramarital relationships in which men are expected to provide material and monetary assistance to their sexual partners; the latter is described as prostitution, and shamed [11,34].

Second, we argue that transactional sex talk reflects *gendered agency, power and control in transactional sex relationships*. Hunter's 2002 study in Mandeni, South Africa explores how certain words are used to elevate men's sexual conquests (*isoka*, a word used to describe conspicuous success with women) while other words are employed to denigrate women with multiple partners (*isifebe*, a word used for both prostitution as well as having multiple partners) ([8]: 108). However, in the same study, women also see multiple partners as a way to gain control over their lives, and this is reflected in their choice of words. Hunter notes: "The very vocabulary of sex - centred, for women, around the verb *qoma *(to choose a man) - is suggestive of women's agency" ([8]: 112). Additional terms reported by young women in different contexts also imply their perceived role as active agents in engaging with men for material or monetary exchange. Men were called 'buzi' (a goat to be milked) in Dar es Salaam, Tanzania [35], and to have transactional sex was 'sengue' (to milk the cow) in Maputo, Mozambique [15]. As a young woman in a township outside of Johannesburg explained:

He is called a chicken because all you want to do with him is get him to give you whatever you want. We say *uyamcutha *[plucking the chicken] ([14] page 26).

In each of these instances the terms utilized by the women participants in these activities denote their perceived or intended role as active initiators of transactional sexual relationships.

Third, we suggest that everyday 'talk' sheds light on *what motivations exist for transactional sexual relationships*. For example, in a review on 'sugar daddies' and 'sugar mamas' (themselves interesting characterisations), Kuato-Defo (2004) suggests that terms used to describe transactional sexual relationships or partners, such as the '4 C's' (i.e. car, cellular phone, cash and clothes) in Swaziland, or 'sponsors' in Ghana (and Madagascar) are an indication that these "practices ... [are] at least in part influenced by an increasingly materialistic society in the context of globalization" [17] (page 15).

The themes presented above also serve as the organizational basis for the presentation of our findings below, and include: 1. talk as a means by which to differentiate appropriate from inappropriate sexual practices within specific worldviews; and 2. gender and agency in sexual exchange relationships. We discuss how everyday talk concerning transactional sex implicates different processes of globalization as a motivation for such practices.

### Study Aims

In this paper we take advantage of a unique opportunity to compare the talk about transactional sex across three study settings: garment workers in Lesotho; young women and men in Antananarivo, Madagascar; and young women and men in Mbekweni, South Africa. By taking as the object of study the talk or discourse around transactional sex, we provide a set of appropriately nuanced contributions to the meaning of transactional sex and to the understanding of gendered power dynamics within these exchange relationships. We then discuss how discourses around transactional sex in our study sites suggest linkages to processes of globalization and carry implications for vulnerability to HIV.

## Methods

### Research Design

The analysis in this article is based on three case studies conducted as part of a multi-country research project that investigated linkages between economic globalization and HIV. The case studies were conducted by independent teams of researchers, each of which was successful in an open, competitive research competition launched in 2007 and co-funded by the Health Economics and HIV/AIDS Research Division (HEARD) in South Africa and the International Development Research Centre (IDRC) In Canada. To be eligible for funding, case studies were required to: empirically investigate a pathway between economic globalization and HIV; be set in a low or middle income country; and have at least one project co-lead who was a researcher from a low or middle income country. Five case studies were awarded funding from a slate of 50 applications. Studies were conducted in 2009-2010. Because the research objectives and methods for each case study were conceived independently from the others, the data resulting from these cases did not necessarily facilitate cross-analysis. However, results relating to discourses around transactional sex emerged in three of the studies (based in Lesotho, Madagascar and South Africa), which motivated analysis of this overarching issue across the studies.

### The Madagascar Case Study

The objective of the case study based in Madagascar was to investigate the relationship between an interest in the consumption of modern goods and sexual behaviour among youth in the urban, peri-urban and rural setting of the capital city of Antananarivo and the urban setting of the northern port town of Antsiranana. Antananarivo is a city of nearly two million that has experienced recent growth both in stores selling luxury goods, as well as in factories where the vast majority of jobs associated with a rapidly expanding textile industry are located (expansion ended with the political crisis in 2009). Data were collected using both qualitative and quantitative approaches between September 2009 and February 2010. A core research team led fieldworkers in data collection that included focus groups with youth and parents of youth, household-based surveys of youth aged 15-24, and follow-up in-depth interviews with a sub-sample of youth survey respondents. The analysis in this article is primarily based on data collected from eight focus group discussions (each including a maximum of six participants) conducted with youth in urban Antananarivo. The objective of these focus groups was to uncover the perceptions of different groups of young people about the meaning and importance of modern goods, and associations, if any, with sexual practices. Participants were selected from two neighbourhoods that reflected the widening gap between rich and poor in Antananarivo. In each neighbourhood participants were convenience-sampled following an effort to determine, through informal interviews with youth and local leaders, the categories of youth most salient to the neighbourhood. Please see Table 1 for a brief description of these focus groups:

**Table 1:** Youth Focus Groups held in Urban Antananarivo, 2009

	**Youth Focus Groups (Participants)**			
	**Wealthier Neighborhood**		**Poorer Neighborhood**	
	**Young Men**	**Young Women**	**Young Men**	**Young Women**
	
University Students	1(n = 6)	1(n = 6)		
High School Students	1(n = 6)	1(n = 6)		
Employed or Looking for work			1(n = 6)	1(n = 6)
"Maditra"*			1 (n = 6)	1 (n = 4)

*"Maditra" loosely translates to naughty or inappropriately behaved. For both women and men included in these focus groups, some of the participants were also university students, but were defined by their local peers as "maditra."

Additional analysis draws on the survey with 625 participants conducted in urban Antananarivo and the 28 in-depth interviews conducted with urban youth respondents.

Contextually, it is important to note the significance of patriarchal Christian belief systems in urban Antananarivo that, alongside neoliberal economic policies, also significantly influence beliefs and restrain behaviours ([33,36,37]. The legacy of missionary involvement beginning in the early 1800s imparted patriarchal moral understandings of sexual behaviour, emphasising women's chastity and marital fidelity [38] but with no parallel moral prescriptions placed on men. As for HIV, prevalence among adults in Madagascar is 0.1%, [39] and indicates that the epidemic is not generalized but rather exists in pockets of vulnerable communities with significantly higher infection levels.

### The South Africa Case Study

The objective of the case study conducted in South Africa was to establish the nature of transactional sex and to explore the intersection between transactional sex and other sexual risk behaviours among young women aged 16-24 years in Mbekweni, a peri-urban community situated 60 kilometers outside of Cape Town. In particular, the study focused on community constructs of modernity, women's power and control in sexual relationships, and links with certain processes of globalization, namely consumerism and the use of modern technology in the maintenance of young people's social networks. Data were collected through focus group discussions, in-depth individual interviews, and key informant interviews. Participants were recruited in Mbekweni using purposive and convenience sampling methods. The analysis in this article is based on the four focus group discussions and seven in-depth individual interviews conducted with young women aged 16-24, and two focus groups conducted with men (one aged 20-39 and one over 40).

Despite the general wealth of the area near Cape Town, Mbekweni is comparatively poor. Its proximity to the wealthier centre of the region continues to determine the realities of community members in Mbekweni in ways that are marked by intra- and interracial social and economic inequalities. Furthermore, the historical circular migration of community residents from the more rural parts of the country means that Mbekweni straddles two cultural identities: a modern, liberal identity, and a traditional, conservative identity. This results in a community with both progressive ideas as well as conservative patriarchal attitudes about women's rights to sex and sexual pleasure.

In the Western Cape, the HIV prevalence rate among women attending antenatal clinics (15-49 years) in 2008 was 16.1% [40]. Furthermore, across South Africa women between 15-24 years (the focus group of this study) are three times more likely to be infected than boys of the same age. A 2010 study in South Africa showed that 14.5% of 15 - 24 year olds had a partner who was more than five years older and that 30.8% of males had reported more than one sexual partner in the 12 months prior to the study [41].

### The Lesotho Case Study

The objective of the case study conducted in Lesotho was to investigate linkages between Preferential Trade Agreements (PTAs), opportunities for economic empowerment, and vulnerability to HIV among garment factory workers in two regions of Lesotho. Both quantitative and qualitative approaches were used; however, the analysis in this article is based on the qualitative data. The qualitative data include 24 in-depth interviews with garment workers (12 women and 12 men) who were employed at a total of six textile factories in Maseru and Maputsoe. Contrary to the case studies conducted in South Africa and Madagascar, issues around transactional sex were not an explicit focus of the study. However, the interview guide included questions exploring participants' experiences since becoming employed at the garment factory with engaging in a relationship or having sex with someone because she/he provided that participant with goods such as food, cosmetics, clothes, cell-phones, transport, school fees, money for rent, somewhere to sleep or cash.

Contextually, in the last two decades the textile and garment sector has become one of Lesotho's primary employers. Furthermore, this industry predominantly employs women (86%). This expansion coincided with a decline in male labour migration to South African mines, and a general decline in male opportunities for employment. The HIV adult prevalence rate is the third highest in the world at 23.2% [39], and, following the trend of the region, women have a significantly higher rate of infection than men. A 2009 study found 41% HIV prevalence among workers in the apparel sector (44.2% among women and 35.6% among men) [42]. The practice of multiple and concurrent partnerships in Lesotho is also very high, exacerbating vulnerabilities to HIV.

### Approach to Analysis in this Study

The present analysis was conducted collaboratively by a research group that included representatives from the three case study teams as well as members of the coordinating team for the overall project. Our approach to analysis was iterative and inductive. The collaborative task of identifying synergies in transactional sex discourses across the case studies began at the launch of data collection for the three studies, and concluded 6-months after the last case study ended. Data analysis involved team members reviewing data related to transactional sex from the three case studies for patterns, surprises and contradictions based on three analytic questions: (1) How is transactional sex framed in this context? (2) How is transactional sex linked with globalization? and (3) What are the implications for vulnerability or resilience to HIV?

## Findings

### The politics of differentiation

Language concerning transactional sex served the purpose of demarcating practices and identities that were and were not deemed acceptable. In order to frame such distinctions, participants' drew on narratives for normative, socially sanctioned identities and practices. A primary example of this is how participants in both Lesotho and Madagascar (where the sampling frames captured a more general population) characterized exchange within the context of sexual and romantic relationships as most often motivated by love.

For instance, a young female textile worker in Lesotho explained that she received financial support in exchange for sex within the context of love:

Yes I have [an exchange] relationship, but it is solely based on love. For example, once my child got ill, and my boyfriend took him to hospital and incurred all the hospital costs, he also gives me money when I ask for it, such as assistance with transport money when I'm broke. And this has been the only relationship in this past year.

Additionally, a 29-year-old man recounted having had a relationship "in which I obtained cash" and "had sex about once in 3 months." He explained that he later married this partner.

In Antananarivo, Madagascar, love was the overwhelming narrative for describing the motivation for gift-exchange in the context of sexual relationships and preceded most conversations detailing less socially acceptable motivations. Participants were asked to discuss whether or not they thought there was a link between gift exchange and sex; in almost every case, participants initially insisted that gifts were offered as part of a loving relationship. Even as women (and some men) identified financial support as a key advantage to having a sexual relationship, they simultaneously rejected the idea that most young people are engaged in relationships *in order to *acquire material possessions. While some men agreed that they might expect sex in return for having offered a gift, for the most part, the narrative was along the lines of the following explanation from a young man from a poor neighbourhood:

For me, it's not because I have given something to a *sipa *(girlfriend) that I expect to get sex. It depends on the two persons' mentality, on the strength of love that links them.

One focus group discussion in Antananarivo was held with young women who their community had characterized as '*maditra' *which translates loosely to 'naughty' or inappropriately behaved. Even for women defined as such, after recounting stories of having had multiple lovers in order to access a better life, these participants argued that a loving relationship should be based on love alone, and that anyone who accepts gifts in explicit exchange for sex is quite simply a prostitute. This insistence is probably at least in part explained by the nature of focus group interviews, which tend to reflect dominant ideologies. The insistence also serves as the basis upon which participants in both South Africa and Madagascar used language to draw boundaries around particular behaviours and to demarcate identities in a way that is consistent with Klesse's (2006) notion of "the politics of differentiation." That is, through their talk about transactional sex, participants drew distinctions between acceptable or desirable behaviours and their alternatives. This differentiation occurred either when discussing their practice of transactional sex as in the South Africa case study, or when discussing their efforts to distance themselves from those whom they perceived to practice transactional sex in the Madagascar case study.

#### *How *uku kura *makes transactional sex acceptable*

In Mbekweni, South Africa, the term '*uku kura' *was used by participants to describe transactional sexual practice in a way that differentiated it from prostitution. While focus group participants and other informants were not able to provide the origins of the term *uku kura*, it certainly enjoyed great popularity in men and women's talk about sexual exploits for material gain in Mbekweni. It is important to note that *uku kura *was not used to describe the exchange of gifts or money in general romantic relationships, which were perceived to be motivated by things other than money or sex. As for those relationships that were believed to be motivated by money or sex: men who buy women alcohol or food at sheebens and taverns with the sole agenda of having sex with them describe themselves as ones who kura women for sex. In the same vain women who pursued men solely for material gain described this practice as *uku kura *men for money. The community understanding of *uku kura *is that women who '*kura' *men for money or goods do not necessarily have sex with these men; this is the important distinction used to differentiate *uku kura *from prostitution, where sex is explicitly exchanged for money. These exchange-based relationships taking place in Mbekweni, namely within *shebeens *or bars, are similar to those described in other parts of South Africa where these practices have also been portrayed as distinct from prostitution (see: [32,43,8,9]). The differentiation hinges on women's insistence that when a woman has managed to *kura *a man (trick or seduce for purposes of getting alcohol, money, etc), the sexual exchange is not necessarily mandatory.

Sometimes you don't have to sleep with the person you are 'kura.' There are ways of running away from him or dumping him.

However, many participants admitted that in reality avoiding having sex with a man you have '*kura'd*' is extremely difficult and rare, particularly because of the real threat of violent retribution for such attempts. The ambiguity created by the possibility of multiple outcomes enables the perception of *uku-kura *as different from commercial sex work and, as such, less stigmatizing. The fact that the practice of *uku kura *is also governed by *shared *implicit (rather than explicit) understandings between the two parties who are engaged the exchange of sex for gifts/money is another differentiating factor that allows it to be perceived differently from prostitution or formal exchanged-based transactions.

To this end, use of the term *uku kura *achieves a paradox of both hiding and revealing what might otherwise be deemed stigmatized behaviour in the study community. Indeed, it is these nuances in the understanding of the term *uku kura *that may enable transactional sex to flourish in Mbekweni.

The subtlety of these nuances is noteworthy. For example, when asked to describe what sex for money or material exchanges entailed, young women participants provided responses such as, "Selling sex for cash", or:

If there was like a freeway that is full of cars from Cape Town, she would go and stand there. *Chippas *[a local and very popular *shebeen*] is full of Cape Town cars so she is going there.

These examples reflect a deliberate, conscious and intentional use of women's sexuality to procure money or materials. Other participants likened the attracting factors (e.g., many cars from the urban centre representing the availability of wealthy men) that characterize the local environment where they go to sell sex (e.g., the bar, *Chippas*) to the 'red light districts' of the city where sex workers sell sex in nearby urban centres, Cape Town and Mfuleni. However, these same women would not permit labelling their practice as prostitution. When asked how this practice is different from prostitution, participants further conflated the two concepts. For example, one participant explained that *uku kura *is not prostitution by stating:

You are not standing on the freeway... . You are not wearing any miniskirts [symbol of prostitution in their judgement].

The subtle distinction in how these women understand *uku kura *compared to prostitution may be too nuanced for easy understanding by an outsider. However, this observation may offer an important message about how young women *own *the terms and meanings that they use to define their sexual behaviour. For a sub-population that has historically had its actions ruthlessly regulated and the meanings of right and wrong defined for them, this might be interpreted as an impressive act of defiance. The mechanisms that have led to young women's apparent sexual and social boldness in this community are not yet clear and present opportunities for further study.

##### *The *mpanataka *the *materialiste *and the *foza orana *in Antananarivo*

In Antananarivo, Madagascar, identities that were associated with transactional sex practice were identities that participants carefully distinguished from their own practice; and made clear efforts to denounce. Below we describe three such identities and practices: *materialiste*, *foza orana *and *manataka*.

Similar in some respects to *uku kura*, participants used the term *manataka*, which means to exploit or swindle someone, from '*manataka paosy*' (to tear open a pocket). Until recently, the connotation applied specifically to women who swindle or exploit men. More recently the term has been applied to both women and men who exploit the opposite sex for their money. Even more specifically, the term also refers to exploitation in order to purchase '*lamaody.' Lamaody *(from the French *la mode*), loosely translates to 'fashionable,' and referred primarily to trendy clothing, the latest technology, and to having a trendy or modern lifestyle.

In the more common conceptualization of *manataka*, participants explained that a girl finds a young man and flirtatiously convinces him to buy her a sought-after *lamaody *item. If the young woman succeeds at this game multiple times, she could be described as a *mpanataka *(one who *manataka*[s] someone). The term itself does not connote sexual exchange; rather, it simply means 'swindle.' However, there is enough implied sexuality that those labelled as *mpanataka *are not well viewed, at the very least because their behaviour is disingenuous. If, however, sex is explicitly exchanged in return, the woman is no longer a *mpanataka*, but a prostitute. As one high school girl explained:

Participant: ...if there isn't any sex at all, then it's like she is just a *mpanataka*. Often there isn't any sex when it's like that, but it depends on the girl...

Interviewer: But what if there is sex?

Participant: Well, then it's the same as her being a *mpivaro-tena *(prostitute).

Unlike Mbekweni, in Antananarivo there is no term that denotes the practice of sexual exchange for goods other than prostitution. Once sex is involved, prostitution is the only label from which to choose. There are, however, additional labels given to women who, as one participant characterized it, "use any means possible" to obtain *lamaody*.

The label '*materialiste*' captures one such identity. This identity describes women, and sometimes men, who seek relationships in order to access money or goods. Male university focus group participants expressed disdain for those who re-arranged their priorities such that material goods trumped the sanctity of women's chastity in Antananarivo. Women who "lay down their body...for the love of *lamaody" *were contrasted with women who engage in exchange relationships for their livelihood (e.g. to obtain school fees). As one young man explained:

...women are becoming nothing more than *materialistes*, ... driven only by money...They materialize their relationships without any consideration for love, self-pride, they just let money prevail ... they go out with someone just to satisfy their needs, ... to be able to keep up with *lamaody *and to get whatever they want to have. ... if the girl does that only for *lamaody*, it's not at all acceptable...if she were doing it to make a living, then I wouldn't mind.

The young man's explanation above echoes many others' words, and could be interpreted as a moral indictment of 'consumption sex,' but a tolerance for 'survival sex.' His ideas reflect the normative view in Antananarivo of women who seek material goods through relationships as pursuing the wrong ends, using the wrong means.

Women who might be identified by others as *materialistes *were even quicker to distance themselves from this identity. After describing a *materialiste *as someone who goes out with someone "not out of love" but in order to "get nice clothing and the like" one university woman focus group participant was then asked if she knew what might influence that practice. Her response illustrates her effort to 'other' this identity:

It's not ... that we, because it seems very disdaining and that's very annoying! ... a university girl should not have such a mentality, but those who do that are women who do not have ... any mental development, and ... are narrow-minded!

Another term in recent increasing circulation among young people in Antananarivo illustrates the sometimes negative connotation ascribed to both material goods as well as the women who use 'inappropriate' means to seek them. The word f*oza orana *- literally a recently discovered invasive species of crayfish - came to refer to items that are deemed to be prevalent and of poor quality. One frequent usage is to describe a cheap cell phone: "mobile *foza orana*."

More recently, the term has been applied to (primarily) women who have multiple sexual partners, or who have sexual partners in order to access money, materials or *lamaody*. The implication being that such women are also both plentiful and 'cheap.' The term *foza orana *is like the other expressions, evolving in its meaning and scope. The dynamic nature of the meaning of the term was perhaps more obvious with *foza orana *because it is such a new concept - the crayfish itself, now a ubiquitous menace, was only discovered in 2006. Upon reflection and discussion, in some focus groups, participants agreed that *foza orana *was an emerging synonym for low-class prostitute. For others, it was an identity that was distinct from prostitution. In summary, *mpanataka *was not always ascribed to women who seek *lamaody*, but appears to be adapting such a connotation; *materialiste *was characterized as a more recent expression, in line with a perceived increasing importance placed on women's quest for consumer goods; and *foza orana *is the newest addition to the lexicon, and appears to characterize the perceived increasing scores of poor women seeking support through sexual exchange. For everyone, these expressions captured identities from which to distance oneself.

##### Gender, agency and power

The terms *uku kura *in South Africa, and *manataka and materialiste *in Madagascar all share an implicit but important assumption: in the relationships or identities denoted by these terms, women exploit men using their sexual power, to their financial gain. What has yet to be made explicit, however, are the even more fundamental underlying realities: women are relying on their sexual prowess largely in the absence of alternative forms of income generation. Furthermore, these practices occur in the context of patriarchal norms and structures that continue to place women in positions that are politically, economically and socially inferior to men. Women in Mbekweni, South Africa, for example, spoke of their conquests and of the sexual pleasure they derived from their relationships with casual lovers or with younger male partners, leaving the impression of themselves as powerful sexual agents. However, when men spoke, and when women described the finer details of their sexual liaisons, it became clear that there were real limits to the agency of these women. Women and men's discourses about how transactional sexual relationships are formed and conducted provide evidence both of women's power as sexual agents, and its limitations.

#### Men's discourse in Mbekweni: Objectifying the 'hunters'

Men who spoke about their involvement in transactional sex in Mbewkeni frequently revealed how they objectify and dehumanize women in these relationships. Overall, young men's talk about young women in the context of transactional sex was characterized by descriptive words such as 'fresh,' 'raw,', 'steak.' The word 'fresh' denotes the preferred younger age category. 'Raw' refers to their preference for sex without condoms. 'Steak' frames young women as a commodity. Two examples use these terms in context:

These older men drink a lot of whisky and brandy so they always want *steak*, they want it raw! Strictly!

This babe is fresh. She's has beautiful thighs, light skin. No, this babe, I am *pouring *into her raw!

Men's description of these sexual exchanges portrayed a picture in which the men were hunted and then seduced by young women. The seduction was described in terms parallel to how one might appraise a piece of meat and, based on the appeal of this product, the men would then be seduced into paying what was necessary to mark this prize. This payment often took the form of buying alcoholic drinks for the women, after which the men explained that they would take charge in determining where, when and how the sexual encounter was to unfold. Men frequently portrayed themselves as in control of a rather unemotional event, as suggested by one male participant's comment, "Then I fucked her and that's it, and then she gets out." Thus, in Mbekweni, men's talk about transactional sex further calls into question women's actual power during these exchanges.

#### Women's discourse in Antananarivo: On gendered-expectations and agency

In Antananarivo, a different set of perspectives was revealed during discussions among different groups of women about gift-exchange within sexual relationships. The issues at their core were commentaries on women's power and autonomy- what women cannot and should not do versus what women can and should do for themselves. For example, in one focus group among young women from a poor neighborhood, a debate was raised over the notion of whether or not a woman can deny sex to a man who has given her a number of gifts:

P3: If he ... like ... buys you stuff, he buys, he buys... and you always take it, and then he asks you...to have sex, and you refuse. You are obliged... In the end, he will force you because you always refuse, you spent his money.

P1: ...but, but, you see, P3, ... you are not obliged to accept and take his gifts! ... You know, it's yours, it's up to you to refuse or to accept!

After two women reflected on the implicit obligation to have sex with a man who has provided you with gifts, a third participant insists that a woman is never obligated to have sex, particularly if she never 'enters the game.' It is contextually important that the women in this exchange were extremely resource-deprived and some had young children, factors that limit real 'choice.' Even so, the notion of whether or not a woman needs such gifts was what distinguished the consensus reached between the focus group of *maditra *women (some of whom were university students) as compared to the (general focus group of) university women. Women identified as *maditra *justified their involvement with multiple partners by referring to women's unequal gendered position in society. The assumption that women cannot and should not get as far ahead as their male counterparts forms the basis of the following young woman's explanation for sexual experimentation with a foreign man:

He can provide everything that I need! Because here, it's not..., because look at the issue! Life is getting much harder. One can no longer cope with it. It doesn't mean that one will misbehave, but just mmm..., one has to think about it... Whatever saves you and allows you to succeed. It's true that you are studying very hard but there's no job either, you're a woman, so you have to get support from a '*vady*' (spouse).

This sentiment that women must rely on men was contested by the university women focus group participants who argued instead that they can and should be self-reliant. Here, women are reflecting on whether or not it is problematic if women seek relationships with men in order to access *lamaody*:

P2: It's not good... because .... you should at least make some efforts for yourself, but should not always wait for the others...

P6: ...you are no longer, you are no longer resourceful. You then become dependent! Dependent on the man. But if you don't do that, you work hard, and have a good job, something acceptable!...and that's it.

This notion of the possibility of self-reliance distinguishes these women's discourses. Women in the *maditra *focus group situated themselves within unequal gendered power relations, and find a creative, yet negatively socially sanctioned path to success through multiple partners and transactional sexual relationships. Women in the latter focus group, however, rejected this premise, suggesting that with their human capital, women can now achieve material success without dependence on men.

This notion was also present among some women in the Mbekweni case study, many of the young women who engage in transactional sex view their ideal world as one where young women are self-reliant and financially independent. However, these notions co-exist with the acknowledgement of a different reality, which sees young women as anything but self-reliant. In light of this tension, the participants of one focus group lamented the effects of transactional sex on their self-esteem, sense of worth and empowerment.

##### Reversals in gendered power

In both Madagascar and Lesotho, rapid economic transformation has led to a hollowing out of more traditional sources of employment for men. In the case of Lesotho, this is particularly apparent with the decline in the mining sector that has taken place almost simultaneous to the rise in employment options for women within the textile industry. Madagascar has seen declines in the benefits to traditional pathways to success through higher education [44]. It is in this context that we can view the emerging evidence in both of these study sites that men are also now engaging in transactional sex as recipients.

This finding is particularly striking among the semi-structured interviews in Lesotho with 12 male and 12 female textile workers. Participants were asked if, since becoming employed at the factory, they had begun a relationship or had sex with someone because of the expectation of being provided with material goods (in particular: food, cosmetics, clothes, cell-phone, transport, school fees, money for rent, and money for tuition, somewhere to sleep, or money). Of the 24 respondents, 2 women and 5 men answered that they had engaged in such transactional arrangements. While the women versus men ratio of positive replies was noteworthy, so was the way in which men and women described these relationships in similar ways using similar terms. For example, one man explained how he acquired money through a sexual relationship:

To tell the truth, it happened. I was expecting that person to provide security so that when need arises she can always help me with the money.

Another male participant explained:

Yes, I used to share ... problems with her. I had two of these partners. I sleep with her once in a month, she provided me with food, clothes, school fees, money for rent, cash.

These accounts suggest that transactional sexual relationships may be serving as a form of social insurance, providing a needed distribution of a small pool of economic capital so as to provide many families with the essentials they need.

In contrast, in Antananarivo there is a term utilized to describe men who are supported financially by women who engage in transactional sex or formal sex work (See [45]). These 'kept' men are labeled *jaombilo*. Relationships between female sex workers and *jaombilo *throw off traditional understandings of gender relations. Men and women participants expressed similar concerns that *jaombilo *disturb the normative gendered-structure in Madagascar and is therefore a socially dangerous identity. As one university man explained:

It's not acceptable if a man does that as it's truly his personality which is at stake. I say that because for us Malagasy, the man is the head [of the family] though if one does that, it's the woman who becomes the guide for everything because [you have become] her property.

The perceived increase in these gender-dynamic reversals are challenging gendered norms and understandings in Madagascar, but they are unlikely to go away as long as the economy remains unstable and continues to expand in low-skilled jobs that cater to women at the expense of higher paid opportunities that once traditionally catered to men.

##### Limitations

In considering these three case studies together, this article offers a more complex view of transactional sex in context and illustrates how similar sets of macro-level inputs interact with unique socio-cultural and political trajectories to generate different realities. It must be re-emphasized, however, that the data in these studies were not intended for direct comparison. The methodologies and goals in each case study were unique.

Although in Mbekweni there is a (perhaps appropriate) sense that transactional sexual behaviour is normative, it is also the case that the methods used to locate research participants actively sought young women and men who were engaged in transactional sexual activities.

The Antananarivo case study, however, sought to assess the extent to which transactional sex was being practiced within a sample loosely representative of the total population of the city. The focus group methodology, used in both cases, is one that tends to excel not at unearthing individual or even collective experience, but rather a sense of normative value systems and understandings. Therefore, the fair amount of 'othering' reflected upon in these focus groups cannot be used to gauge actual prevalence of behaviour. That said, within Antananarivo, the survey data support the assertion that very few women engage in formal transactional sex or multiple sexual partnerships (0.80% and 5.5%, respectively).

The case of Lesotho is different in that it did not set out to understand transactional sex at all, yet participants raised this issue in the context of broader discussions. It is therefore important to resist extrapolating these findings to generalizable distinctions between the countries or the study sites incorporated in this work. It is equally important to recall that the discourses captured here reflect a specific point in time and are extremely dynamic. The term *foza orana*, for example, is in evolution; the identities it reflects are constantly being re-negotiated.

## Discussion

### How transactional sex talk reflects on globalization

The everyday talk referring to various identities and practices described above reflect the more recently described paradigm linking transactional sex and globalization through consumption, whereby young women engage in transactional sex in order to consume modern goods, guided by increasing importance placed on consumerism in so many societies. The framing of the identities described in both Madagascar and South Africa further support this literature.

*Uku kura *was described by the women who participated in these exchanges as rooted in social survival, the importance of keeping materially in step with their peers. The *materialiste *identity reflects on the perceived outside influence of both the goods being sought after and the behaviors they may or may not be encouraging. Significantly, the term itself is French, from outside of Madagascar, like the goods that individuals defined as such are seeking. The notion that money and material motivates these kinds of exchanges is reflected in popular culture in Lesotho, in the following lyrics of a popular local *famo *band:

Women fall in love with young boys (basali ba ratana le bashanyana)

Men fall in love with young girls (banna ba ratana le banana)

All in the name of money (lebitsong la chelete)

Participants in Madagascar perceived such linkages between the outside world and the moral compass of their peers. Some participants rooted youths' current obsession with material goods in globalization, referring to the perceived need to have fashion as being "caught by globalization." Similarly, some explained youth's engagement in multiple partners and reliance on relationships in order to access goods as evidence that the "mind has been destroyed by globalization." These findings echo previous studies highlighting how perceived changes in morality are blamed on new cultural influences from outside [46];[47].

Such reflections on how Western goods and lifestyles impact local desires could be summarized as indicative of cultural processes of globalization. Indeed, there are also indications,--reflected in transactional sex talk-of processes of economic globalization influencing transactional sexual practices. For example, the term *foza orana *refers to both the flood of the stuff available under liberalized trade and the flood of young women having to rely on their sexuality to get by. The juxtaposition of increased availability of items and low spending power was not lost on youth participants. As one young man expressed:

For a developing country like ours, trying to keep up with other countries does not do us any good because we don't have the same purchasing power, we don't have the same standard of living as those countries.

In addition, the gendered-reversals present in Madagascar and Lesotho point to a different set of processes that can also be described as related to economic processes of globalization. The hollowing out of traditional sources of employment for men, accompanied by unstable economic environments, has also been located as having an impact on women's and increasingly, men's perceived increasing reliance on transactional sex [13,46]. These linkages between different processes of globalization and transactional sex, carry, in turn, implications for vulnerability to HIV.

### Silences, symbolic distancing and changing gender dynamics: Implications for HIV vulnerability

This set of studies did not seek to address the specific relationship between transactional sex and discourses and practices concerning HIV risk and risk reduction, although in South Africa HIV risk was a more active focus of enquiry. Nevertheless, in none of these studies did participants' concerns about HIV appear to guide their practices. In most cases, rather, the norm was silence concerning HIV. In the hyper-epidemic contexts of Southern Africa, such silence may be interpreted in at least two different ways: (1) as some participants phrased it, HIV was 'peanut butter,' ubiquitous, the underlying attitude being that 'you can do nothing about it;' this implies a normalization of infection and fatalism, to the point of acting as if individual sexual behavior does not affect HIV risk; (2) alternatively, association of HIV risk with formal sex work or promiscuity, from which respondents carefully distance themselves, so that their practices, albeit transactional, are still 'safe' or 'okay.'

Concerning the first point, potentially close relationships with many peers known to be living with (and now also treated for) HIV, and yet failure to rationally associate infection with specific practices might be explained by a conceptualization of HIV infection as highly likely and possibly inevitable. Such a reading implies a particular way of normalizing the epidemic in Southern Africa; people go on with their regular sex life and act upon the consequences of infection if they must. Normalizing of HIV has also been described in the very different context of gay men in North America and Western Europe [48-50], where likelihood of infection is acknowledged not always with fear [51], in fact, there are accounts of a certain eroticization of semen exchange and possibly of infection per-se, with people actually seeking to get infected [52]. Perhaps normalizing of HIV represents a form of coping and resilience vis-à -vis HIV in both the high prevalence context of Southern Africa and the high prevalence context of urban gay communities in North America/Europe, where people assume HIV risk by treating it as inevitable or by transforming their sexual lives through systematic references to HIV and even assigning erotic value to the risk of infection.

Regarding the second point, these case studies demonstrate how participants use language to actively differentiate their behaviors from those that they deem inappropriate, which are precisely related to formal sex work (and its HIV risk implications). In Mbekweni, *uku kura*, or informal sex for money exchange, was differentiated from prostitution. In the case of Antananarivo, identities such as the *mpanataka*, the *foza orana *or the *materialiste*, where the intentions are money as opposed to love were deemed akin to prostitution and, therefore, shunned. As such, participants distanced themselves from these identities even if they practice the behaviors implied by such labels. Juxtaposing the bases upon which identities were differentiated in Mbekweni with Antananarivo suggests a contradictory set of possible implications for vulnerability to HIV in these study sites. While much higher HIV prevalence in the context of Mbekweni coupled with the acceptance of transactional sex (as compared to prostitution) implies greater potential for high risk behavior in Mbekweni, this open acceptance of transactional sexual practice provides an opportunity for open dialogue on the risk of transactional sexual practices. The reverse set of concerns exists in Antananarivo where transactional sexual practices are shunned, a framing which may, overall, discourage their practice; however, behavioral indicators suggest that such exchange relationships are slowly increasing; and if they remain unacceptable, they will be practiced 'underground' rendering dialogue on sexually transmitted infection prevention in this context more difficult. Other literature from Southern Africa and Madagascar has described how condom use can be compromised in transactional sexual relationships, which, because of the nuanced meaning of these sexual exchanges, exist outside of the realm of more conventional messaging targeting sex workers [53-55].

One third issue deserves discussion: the implications of these informal transactional sex practices for women's agency and HIV vulnerability. In the Antananarivo and Mbekweni cases, the identities and activities described by young women and men provide additional support for the growing body of literature that challenges assumptions of women as passive victims within the context of informal sexual exchange ([8,14,15,26]). Rather, to *kura *a man in Mbekweni or to *manataka *a man in Antananarivo both imply that women act as powerful sexual agents, utilizing their sexuality toward locating and then extracting money or material goods or favours from a partner of their choosing. However, our findings, particularly from Mbekweni, also support evidence that suggests that women continue to operate within highly unequal gendered power structures. Furthermore, these power structures are most clearly defined at the point of the sexual encounter, where men typically determine the terms and, in some cases, do so with violence ([15,43]). All three points described above deserve further exploration through new, specific studies that identify implications for HIV prevention. Nevertheless, potential avenues for action can be briefly pointed out. First, if new evidence is found to support the hypothesis of resilience through fatalistic acceptance of HIV risk, then substantial education and communication work is needed to strengthen alternative views of HIV that, without reinforcing stigma or anxiety, revitalize HIV awareness in the general population (particularly among youth) and normalize preventive strategies. Second, some evidence already supports the idea that participants view informal transactional sex as very distinct from prostitution and hence not risky. Assuming that programmes targeted at sex workers are reaching these other populations engaged in transactional sex would be a tragic mistake (see [53]; [15]). Interventions need to grapple with these more nuanced identities and the specific social contexts in which they are produced and transformed, and direct attention to these kinds of exchanges as different from, but as significant as formal sex work (see also [15,43]). Third, it appears that negotiation of sex for material exchange in a non-explicit way reduces women's power to insist upon condoms. Our evidence presented here regarding the limits on women's power within transactional sexual relationships further supports the need for women to have options toward economic independence outside of their sexuality.

## Conclusion

While studies have in the past carefully examined the practice of transactional sex and detailed the terms used to describe that practice in context; no study has taken as its object of study 'talk' about transactional sex and related sexual practices. By focusing on everyday talk and the terms used to denote transactional sex, we locate not only definitions of transactional sex, but how talk about terms used to describe transactional sex is morally framed for people within their local context; and how in many cases these terms represent identities that people wish to differentiate from themselves. We have taken advantage of an opportunity to comparatively explore such talk across three different study sites. Through this unique analysis we have contributed to a better understanding of both emerging sexual practices and their implications for HIV vulnerability. First, we add to an understanding of the motivations behind these transactional sexual practices, rooted in many cases in notions of 'globalization,' often depicted as an influx of goods without an equivalent increase in spending power. We also draw further attention to the value of the concept of transactional sex as one that is distinct from sex work from the point of view of its participants and therefore one that must remain distinct from the point of view of intervention efforts as well. Finally, we illustrate a challenge for such prevention efforts in reflecting on the paradoxical relationship between the gendered power implied in talk about sexual relationships compared to the gendered power described when recounting sexual practices. While women talk about exploitatively extracting financial resources from a man of their choosing, they often do so on that man's terms.

We concur with Mark Hunter's assessment that "... Although with its own obvious limitations, the concept [of transactional sex] is nevertheless especially useful in informing AIDS policy interventions such as 'education' campaigns that can often neglect underlying social relations such as those reflected and constituted by gifts" ([8]:101). Our work suggests that transactional sex needs to continue to be reflected in the literature as it is perceived, something very different from, but of at least equal concern to formal sex work in the efforts to curb HIV transmission.

## Competing interests

The authors declare that they have no competing interests.

## Authors' contributions

All Authors have read and approved the final manuscript. KS coordinated the writing of this paper, led the conception of the analysis, wrote the majority of the manuscript, and led the data collection, analysis and writing of the Madagascar case study. SN coordinated the overarching study, helped conceive of this analysis, and drafted sections of the manuscript. CR assisted in the coordination of the overarching study, helped conceive of this analysis, and drafted sections of the manuscript. SW helped conceive of the analysis, contributed to data interpretation, and helped draft sections of the manuscript. YZ co-conceptualized, designed, collected, analyzed and interpreted the data for the SA case study, and contributed to analysis for this paper. TT supervised data collection for the Lesotho case study, contributed to analysis for this paper, and drafted part of the Lesotho section for this paper. PT contributed to execution of the Lesotho case study, contributed to the analysis of this paper, and draft part of the Lesotho section for this paper. HMB conceptualized and coordinated the Lesotho case study until January 2010, and contributed to development of this paper. CC contributed to the analysis of this paper and drafted much of the Discussion section. LT co-conceptualized and assisted with the analysis of the data for the South African case study, and contributed to development of this paper. PGR coordinated the qualitative data collection for the Madagascar case study, conducted many interviews, and contributed to the analysis, which was used in developing this paper. RZ co-coordinated the qualitative data collection for the Madagascar case study, conducted many interviews, and contributed to the analysis, which was used in developing this paper.

## Authors' information

All authors are involved in the HEARD- and IDRC-funded Economic Globalization and HIV/AIDS multi-country study that aimed to contribute a better understanding of the relationship between globalization and AIDS through five case studies. Some authors are primarily affiliated with a specific case study (YZ, LT- South Africa; KS, RZ, PGR- Madagascar; TT, PTT and HMB- Lesotho); while others are affiliated with the broader project (SN, CR, SW and CC). Together, we recognized an important synergy in the findings emerging from at least three of the five case studies with regards to transactional sex, and how it was framed. This paper brings together the expertise of advisors to the overall project along with individual case study contributors to draw out these findings.

## References

[B1] Dunkle KL Jewkes RK Brown HC Gray GE McIntryre JA Harlow SD Transactional sex among women in Soweto, South Africa: prevalence, risk factors and association with HIV infection Social Science & Medicine 2004 59 1581 1592 10.1016/j.socscimed.2004.02.003 15279917

[B2] Luke N Kurz KM Cross-generational and Transactional Sexual Relations in Sub-Saharan Africa: Prevalence of Behavior and Implications for Negotiating Safer Sexual Practices 2002 Washington, D.C., AIDSMark, USAID Project

[B3] Halperin DT Epstein H Concurrent sexual partnerships help to explain Africa's high HIV prevalence: implications for prevention Lancet 2004 364 4 6 15234834 10.1016/S0140-6736(04)16606-3

[B4] Farmer P AIDS-Talk and the Constitution of Cultural Models Soc Sci Med 1994 38 801 809 10.1016/0277-9536(94)90152-X 8184331

[B5] Kaler A AIDS-talk in everyday life: the presence of HIV/AIDS in men's informal conversation in Southern Malawi Soc Sci Med 2004 59 297 10.1016/j.socscimed.2003.10.023 15110420

[B6] Ho J From Spice Girls to Enjo-Kosai: Formations of Teenage Girls' Sexualities in Taiwan Inter-Asia Cultural Studies 2003 4 325 336 10.1080/1464937032000113033

[B7] Dunkle KL Jewkes R Nduna M Jama N Levin J Sikweyiya Y Transactional sex with casual and main partners among young South African men in the rural Eastern Cape: Prevalence, predictors, and associations with gender-based violence Social Science & Medicine 2007 65 1235 1248 10.1016/j.socscimed.2007.04.029 17560702 PMC2709788

[B8] Hunter M The Materiality of Everyday Sex: thinking beyond 'prostitution' African Studies 2002 61 99 120 10.1080/00020180220140091

[B9] Preston-Whyte E Varga C Oosthuizen H Roberts R Blose F Parker R, Barbosa RM, Aggleton P Survival Sex and HIV/AIDS in an African City Framing the Sexual Subject: The Politics of Gender, Sexuality, and Power 2000 Berkeley, CA: University of California Press 165 191

[B10] Mill JE Anarfi JK HIV risk environment for Ghanaian women: challenges to prevention Soc Sci Med 2002 54 325 337 10.1016/S0277-9536(01)00031-4 11824910

[B11] Romero-Daza N Multiple Sexual Partners, Migrant Labor, and the Makings for an Epidemic: Knowledge and Beliefs about AIDS among Women in Highland Lesotho Human Organization 1994 53 192 205

[B12] De Vogli R Birbeck GL Potential Impact of Adjustment Policies on Vulnerability of Women and Children to HIV/AIDS in Sub-Saharan Africa Journal of Health Population and Nutrition 2005 23 105 120 16117362

[B13] Cole J Fresh contact in Tamatave, Madagascar: Sex, money, and intergenerational transformation American Ethnologist 2004 31 573 588 10.1525/ae.2004.31.4.573

[B14] Selikow T-A Bheki Z Cedras E The Ingagara, the Regte and the Cherry: HIV/AIDS and Youth Culture in Contemporary Urban Townships Agenda 2002 53 22 32

[B15] Hawkins K Price N Mussa F Milking the cow: Young women's construction of identity and risk in age-disparate transactional sexual relationships in Maputo, Mozambique Global Public Health 2009 4 169 182 10.1080/17441690701589813 19333807

[B16] Kaufman CE Stavrou SE 'Bus fare please': the economics of sex and gifts among young people in urban South Africa Culture Health & Sexuality 2004 6 377 391 10.1080/1369105041000168049221971187

[B17] Kuato-Defo B Young People's Relationships with Sugar Daddies and Sugar Mummies: What do we know and What do we need to know? African Journal of Reproductive Health 2004 8 13 37 10.2307/3583175 15623116

[B18] Bene C Merten S Women and fish-for-sex: Transactional sex, HIV/AIDS and gender in African fisheries World Development 2008 36 875 899 10.1016/j.worlddev.2007.05.010

[B19] Leclerc-Madlala S Age-disparate and intergenerational sex in southern Africa: the dynamics of hypervulnerability AIDS 2008 22 S17 S25 19033752 10.1097/01.aids.0000341774.86500.53

[B20] Norris AH Kitali AJ Worby E Alcohol and transactional sex: How risky is the mix? Social Science & Medicine 2009 69 1167 1176 10.1016/j.socscimed.2009.07.015 19713023

[B21] Day S Prostitute Women and AIDS: anthropology AIDS 1988 2 421 428 10.1097/00002030-198812000-00002 3149489

[B22] de Zalduondo B Prostitution Viewed Cross-Culturally: Toward Recontextualizing Sex Work in AIDS Intervention Research The Journal of Sex Research 1991 28 223 248 10.1080/00224499109551607

[B23] Fordham G A New Look at Thai AIDS: Perspectives from the Margin 2004 New York: Berghahn Books

[B24] White L The Comforts of Home: Prostitution in Colonial Nairobi 1990 Chicago: The University of Chicago Press

[B25] Wardlow H Anger, economy, and female agency: Problematizing "prostitution" and "sex work" among the Huli of Papua New Guinea Signs 2004 29 1017 1040 10.1086/382628

[B26] Leclerc-Madlala S Transactional sex and the pursuit of modernity Social Dynamics-A Journal of the Centre for African Studies University of Cape Town 2003 29 213 233

[B27] Swidler A Watkins S Ties of Dependence: AIDS and Transactional Sex in Rural Malawi Studies in Family Planning 2007 38 147 162 10.1111/j.1728-4465.2007.00127.x 17933289

[B28] Nshindano C Maharaj P Reasons for multiple sexual partnerships: perspectives of young people in Zambia Ajar-African Journal of Aids Research 2008 7 37 44 10.2989/AJAR.2008.7.1.5.433 25871270

[B29] Smith DJ "These girls today na war-o": Premarital sexuality and modern identity in southeastern Nigeria Africa Today 2001 47 98 120

[B30] Leap WL Boellstorff T Speaking in Queer Tongues: Globalization and Gay Language 2004 Urbana and Chicago: University of Illinois Press

[B31] Klesse C Polyamory and its 'Others': Contesting the Terms of Non-Monogamy Sexualities 2006 9 565 583 10.1177/1363460706069986

[B32] Wojcicki JM Commercial sex work or ukuphanda? Sex-for-money exchange in Soweto and Hammanskraal area, South Africa Culture Medicine and Psychiatry 2002 26 339 370 10.1023/A:1021291922026 12555904

[B33] Stoebenau K Symbolic capital and health: The case of women's sex work in Antananarivo, Madagascar Social Science & Medicine 2009 68 2045 2052 10.1016/j.socscimed.2009.03.018 19362403

[B34] Spiegel AD Spiegel AD, McAllister PA Polygyny as myth: Towards Understanding Extramarital Relations in Lesotho Tradition and transition in Southern Africa: Festschrif for Philip and Iona Meyer 1991 London, U.K.: Transaction Publishers 145 166

[B35] Silberschmidt M Rasch V Adolescent girls, illegal abortions and "sugar-daddies" in Dar es Salaam: vulnerable victims and active social agents Social Science & Medicine 2001 52 1815 1826 10.1016/S0277-9536(00)00299-9 11352408

[B36] Rakotomalala MM Contribution à une anthropologie de la sexualité Merina (Centre de Madagascar) 1996 Institut National des langues et civilisations orientales21972277

[B37] Raison-Jourde F Bible et pouvoir à Madagascar au XIXe siècle: invention d'une identité chrétienne et construction de l'Etat (1780-1880) 1991 Paris: Karthala

[B38] Predelli LN Sexual control and the remaking of gender: The attempt of nineteenth-century Protestant Norwegian women to export Western domesticity to Madagascar Journal of Womens History 2000 12 81 103 10.1353/jowh.2000.0044

[B39] UNAIDS WHO AIDS Epidemic Update, 2008. -98 2008 Geneva, Switzerland, UNAIDS

[B40] South Africa National Department of Health National Department of Health: National Antenatal and Syphilis Prevalence Survey 2008

[B41] Kibel M Lake L Pendlebury S Smith C The South African Child Gauge 2009/2010 2010 Cape Town, Children's Institute, University of Cape Town

[B42] Wallergren K Follow-up HIV Sero-Prevalence Study Report, October 2009 2009 Apparel Lesotho Alliance to Fight AIDS (ALAFA) 1 81

[B43] Wojcicki JM "She drank his money": Survival sex and the problem of violence in taverns in Gauteng Province, South Africa Medical Anthropology Quarterly 2002 16 267 293 10.1525/maq.2002.16.3.267 12227257

[B44] The World Bank The World Bank Group: World Development Indicators Online The World Bank 2008 2

[B45] Cole J The Jaombilo of Tamatave (Madagascar), 1992-2004: Reflections on Youth and Globalization Journal of Social History 2005 38 891 914 10.1353/jsh.2005.0051

[B46] Rodlach A Witches, Westerners, and HIV: AIDS and cultures of blame in Africa 2006 Walnut Creek, CA: Left Coast Press

[B47] Goldstein D Once upon a virus: AIDS Legends and Vernacular Risk Perception 2004 Logan, Utah: Utah State University Press

[B48] Tomso G Viral Sex and the Politics of Life South Atlantic Quarterly 2008 107 265 285 10.1215/00382876-2007-066

[B49] Tomso G Bug Chasing, Barebacking, and the Risks of Care Literature and Medicine 2004 23 88 111 10.1353/lm.2004.0014 15264511

[B50] Rosenbrock R Dubois-Alber F Moers M The normalization of AIDS in Western European Countries Social Science & Medicine 2000 50 1607 1629 10.1016/S0277-9536(99)00469-4 10795967

[B51] Flowers P Duncan B Knussen C Re-appraising HIV testing: An exploration of the psychosocial costs and benefits associated with learning one's HIV status in a purposive sample of Scottish gay men British Journal of Health Psychology 2003 8 179 194 10.1348/135910703321649150 12804332

[B52] Crossley M Making sense of 'barebacking': Gay men's narratives, unsafe sex and the 'resistance habitus' British Journal of Social Psychology 2004 43 225 244 10.1348/0144666041501679 15285832

[B53] Stoebenau K Hindin MJ Nathanson CA Rakotoarison PG Razafintsalama V "...but then he became my *sipa*": The Implications of Relationship Fluidity for Condom Use among Women Sex Workers in Antananarivo, Madagascar American Journal of Public Health 2009 99 811 819 10.2105/AJPH.2007.118422 19299685 PMC2667855

[B54] Voeten HACM Egesah OB Varkevisser CM Habbema JDF Female sex workers and unsafe sex in urban and rural Nyanza, Kenya: regular partners may contribute more to HIV transmission than clients Tropical Medicine & International Health 2007 12 174 182 17300623 10.1111/j.1365-3156.2006.01776.x

[B55] Manuel S Obstacles to condom use among secondary school students in Maputo city, Mozambique Culture Health & Sexuality 2005 7 293 302 10.1080/13691050412331321302 16864204

